# Lipidomic Profiling of Adipose Tissue Reveals an Inflammatory Signature in Cancer-Related and Primary Lymphedema

**DOI:** 10.1371/journal.pone.0154650

**Published:** 2016-05-16

**Authors:** Lisa M. Sedger, Dedreia L. Tull, Malcolm J. McConville, David P. De Souza, Thusitha W. T. Rupasinghe, Spencer J. Williams, Saravanan Dayalan, Daniel Lanzer, Helen Mackie, Thomas C. Lam, John Boyages

**Affiliations:** 1 Department of Clinical Medicine, Faculty of Medicine & Health Science, Macquarie University, Sydney, NSW, Australia; 2 Metabolomics Australia, Bio21 Institute, The University of Melbourne, Melbourne, VIC, Australia; 3 School of Chemistry, The University of Melbourne, Melbourne, VIC, Australia; 4 Daniel Lanzer Clinic, Malvern, Melbourne, VIC, Australia; 5 Macquarie University Hospital, North Ryde, Sydney, NSW, Australia; Fundação Oswaldo Cruz, BRAZIL

## Abstract

Cancer-related and primary lymphedema (LE) are associated with the production of adipose tissue (AT). Nothing is known, however, about the lipid-based molecules that comprise LE AT. We therefore analyzed lipid molecules in lipoaspirates and serum obtained from LE patients, and compared them to lipoaspirates from cosmetic surgery patients and healthy control cohort serum. LE patient serum analysis demonstrated that triglycerides, HDL- and LDL-cholesterol and lipid transport molecules remained within the normal range, with no alterations in individual fatty acids. The lipidomic analysis also identified 275 lipid-based molecules, including triacylglycerides, diacylglycerides, fatty acids and phospholipids in AT oil and fat. Although the majority of lipid molecules were present in a similar abundance in LE and non-LE samples, there were several small changes: increased C20:5-containing triacylglycerides, reduced C10:0 caprinic and C24:1 nervonic acids. LE AT oil also contained a signature of increased cyclopropane-type fatty acids and inflammatory mediators arachidonic acid and ceramides. Interestingly C20:5 and C22:6 omega-3-type lipids are increased in LE AT, correlating with LE years. Hence, LE AT has a normal lipid profile containing a signature of inflammation and omega-3-lipids. It remains unclear, however, whether these differences reflect a small-scale global metabolic disturbance or effects within localised inflammatory foci.

## Introduction

Lymphedema (LE) is a condition where lymphatic fluid accumulates within interstitial tissues causing swelling. In developed countries LE most often occurs as a consequence of, or “secondary to”, treatment for cancer. Cancer patients most at risk of developing cancer related LE include patients with breast cancer who require lymph node staging or treatment (sentinel node biopsy plus/minus dissection and/or axillary radiation, and often with chemotherapy) due to cancer metastasis [1]. Indeed conservative estimates suggest that up to 28% of such patients in developed countries will develop arm LE subsequent to their breast cancer treatment [1]. Similarly, up to 75% of head and neck cancer patients can develop throat, facial, neck or internal LE [2] and a third of pelvic cancer patients will develop lower limb LE within 1 year of surgery [3]. Hence, cancer-related LE is widely accepted as one of the most frequent long-term complications of cancer treatment. Primary LE can also occur spontaneously, usually presenting first during childhood, and often with a genetic origin [4] or resulting from non-cancer-related tissue injuries. Hence LE occurs due to compromised lymphatic drainage that may be directly caused by, or contributed to, by injury or genetic alterations that lead to defects in lymphatic vessel biogenesis or malformations in lymph vessel valve formation and reduced lymphatic function [4]. Unfortunately, there is currently no cure for either primary or secondary (cancer-related) LE. Thus, LE is a chronic condition that impacts on a person’s ability to live a normal life because the condition tends to worsen over time and physical disfigurement and functional disability impacts on quality of life [5,6].

One of the major problems associated with chronic LE is that the affected tissues frequently become consolidated with adipose tissue (AT) [7,8]. Once this occurs compliance to conventional treatments comprising compression, bandaging and massage often declines as it becomes evident that they cannot reduce the LE-associated AT that then predominates within the affected area. Fortunately, there is a surgical treatment option for people living with advanced AT rich LE: liposuction surgery [9–12] which physically removes the AT that predominates the affected area [7,8]. Although this treatment is highly beneficial for LE patients, immediately reducing the LE affected limb size, the reasons for the production of LE AT are still emerging and the LE pathology is incompletely understood [13]. Thus, although it is clear that adipogenesis normally involves cytokines, hormones, transcription factors, and miRNAs that temporally regulate gene expression to drive the maturation of pre-adipocytes into mature lipid-containing adipocytes [14,15], yet very little is currently known about adipogenesis within the LE AT itself. Nothing is known, for example, about the kinds of lipid-based molecules that are produced by LE tissue adipocytes. Interestingly, AT is actually integral to the mammalian immune system and, in fact, lymph nodes normally exist in close physical association with AT, where lipolysis liberates free fatty acids and lipid-mediators that fuel immune system cells [16]. Furthermore, while cholesterol is normally transported in blood via lipoproteins, either as cholymicrons, very low-density lipoproteins (VLDL), intermediate, or high-density lipoproteins (HDL), transport of lipids back from cells and tissues to the liver occurs via HDL and lymph–a process known as reverse cholesterol transport [16]. Here, efflux of cellular cholesterol is released into intracellular space though the combined actions of ABC transporters, scavenger receptor-B1, Cyp27A1, caveolin, or passive diffusion (each mechanism varying depending in part on cell type) [17], where it is ultimately removed by HDL and trafficked back from the interstitium through the lymphatics (for a recent review see [18]). Thus, lipid transport is necessarily integral to LE because lymphatic drainage is altered when lymph nodes are removed, or when lymphatic vessels and valves are damaged or malfunction. Indeed, recent animal-based studies suggest impaired reverse cholesterol transport occurs in experimentally-induced murine LE [19]. If there are defects in reverse cholesterol transport in human LE then the extent to which lipid metabolism is altered or imbalanced are unclear. So, too, little is known about the consequences of chronic LE swelling on serum lipid level and lipid-associated lipoproteins in human LE. Therefore to begin to better understand the pathobiology of AT production in advanced LE we examined the serum lipids and experimentally generated the first AT lipid profiles of advanced cancer-related LE patients and compared them with normal serum, and to anatomically-matched arm and leg normal, non-LE, AT.

## Materials and Methods

### Human Research Ethics Approval

This study was reviewed and approved by the Macquarie University Human Research Ethics Committee (HREC) prior to its initiation. All elements of the study were subsequently conducted according to the HREC approved protocols, such that all normal tissue donors, and patients with LE, donated their blood and AT liposuction samples voluntarily and with informed written consent.

### Patient and Healthy Control participants study inclusion criteria

**Fig 1:**
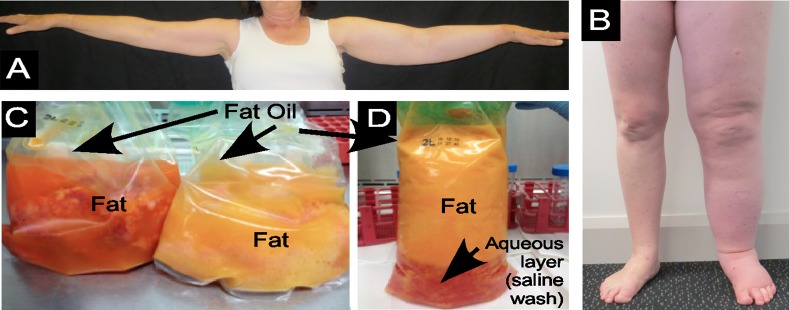
LE and lipoaspirate tissue samples. (A) Left arm LE, (B) Left leg LE, (C) LE AT liposuction aspirate and (D) Normal non-LE (cosmetic surgery) AT liposuction aspirate, showing presence of liquid oil (Oil) and solid fat (Fat). Also evident is the aqueous layer of the saline wash within the cosmetic surgery aspirate.

All patients with LE (n = 15, Table 1) were seeking medical assessment and treatment for advanced limb LE at the Advanced Lymphedema Assessment Clinic at Macquarie University Hospital. These patients usually presented with a limb volume difference of at least >20–25% and are offered liposuction surgery based on the presence of LE-associated AT as determined primarily by a “non-pitting” LE presentation [8,20] and confirmed by magnetic resonance imaging [9]. Thus liposuction surgery is a treatment option for patients who have experienced negligible recent benefit from conventional LE treatments due to the consolidated build up of substantial amounts of AT. For comparison to normal tissue we also obtained healthy human arm and leg (limb) AT (n = 7; Table 1). This tissue was obtained from cosmetic surgery patients who had opted for liposuction surgery at a private Melbourne cosmetic surgery clinic for undeclared personal reasons. An example image of the limb LE patient and the LE and non-LE lipoasparates is shown (Fig 1). All LE patients and healthy donors also provided their weight and height measurements, which were used to calculate the individual’s body mass index (BMI) and thereby to enable identification and exclusion of study participants who might would otherwise not be classified as having a ‘normal BMI’, we according to the Heart Foundation of Australia’s classification scale (http://heartfoundation.org.au/). Thus, all study participants were within normal BMI normal range (15<25), except for two LE patients who had a pre-surgery body mass index (BMI) <30 kg/m^2^. However, these two LE patients were not considered to be obese because the BMI of one patient returned to <30 kg/m^2^ quickly after limb liposuction, and the other has primary leg LE affecting both limbs i.e., clearly these patients pre-surgical data reflected their lymphedematous limbs rather than a condition of generalized body obesity. It was also evident that several individuals in both LE and non-LE cohorts were “healthy but overweight” (BMI = 25–29) consistent with many current Western country demographics (individual data not shown). A statistical analysis of the cohort characteristics data, specifically age, gender and BMI, was performed via a standard Student’s T test with no assumption for data distribution normality given the relatively small sample sizes (Table 1).

**Table 1:** Study cohort characteristics.

Cohort Characteristic	x (range)	*p* value^1^
**Lymphedema AT donors (n = 15)**		
Mean age (years)	49.3 (18–68)	
Gender (male/female)	0/15	
Mean BMI	27.1 (19–37)^2^	
Mean years living with LE	4.8 (2–29)	
Primary LE cases	3	
Secondary LE cases	12	
**Normal AT donors (n = 7)**		
Mean age (years)	26.7 (18–50)	0.0018*
Gender (male/female)	1/6	
Mean BMI	23.4 (21–27)	0.0226*0.0692^3^
**Normal blood donors (n = 11)**		
Mean age (years)	38.9 (27–58)	0.0680
Gender (male/female)	1/13	
Mean BMI	24.8 (21–29)	0.2289

^1^ *p* value (two-tailed t-test, control versus LE)

(*) p <0.05.

^2^ Two LE BMI values are >30 (31 and 37). After removing these two patient samples the revised LE cohort sample rage is 19–29

^3.^ The t-test was re-calculated minus two LE BMI values >30. The new p >0.05 indicates the similarity to the normal cohorts.

### Sample collection and tissue processing

Liposuction surgery for cancer-related LE was performed under general anaesthesia at Macquarie University Hospital by author TL who was specially trained by Professor Brörson and Professor Munnoch, who have developed what is now a well-established procedure for advanced LE [10–12,21]. The surgery was performed on LE-affected arms and legs (Fig 1A and 1B) after the application of a tourniquet. Briefly, subcutaneous AT was removed through multiple small incisions located along the length of the affected limb, via a Helixed Tri Port III cannula (usually 22–30 cm long and 4–5 mm wide) connected to a vacuum pump [9]; the same procedure has now been employed successfully internationally for more than a decade [10–12]. The extracted arm or leg LE AT lipoaspirate (Fig 1C) was immediately transported to the Faculty of Medicine and Health Research Laboratories, processed as described below and stored at -80°C in a AT Sample Bank generated by LMS. Healthy human limb (arm or leg) AT was collected with the donor’s informed consent, at a private cosmetic surgery clinic, Dr Lanzer Clinic, Melbourne, from clients opting to undergo liposuction surgery. Briefly, a healthy lipo-aspirate tissue (Fig 1D) was obtained by standard plastic surgery procedures under general anaesthesia. After small incisions were made, the surrounding area was filled with Klein tumescent fluid using 20 gauge needle, before applying suction and subsequent extraction of AT via a 14 gauge Klein microcannula of the capastrano variety [22]. Cosmetic liposuction surgery patients then wear a compression binder continuously for approximately 2 weeks, then intermittently for 2 weeks post-operatively, in order to minimize the potential for adverse events or post-surgical complications [23].

The LE and normal (cosmetic) liposuction surgery AT lipoaspirates (Fig 1C and 1D) were poured into several 50 ml Falcon tubes and centrifuged at approximately 350 g for 5 minutes to separate the liquid “fat oil” from the solid intact AT i.e. intact adipocytes otherwise referred to here as AT “fat”. The remaining underlying aqueous tissue components were also collected, and the blood erythrocyte pellet was discarded. After centrifugation, the oil was removed and stored frozen at -80°C. Next, the intact adipocytes, and aqueous layers, were carefully removed, separately, and similarly placed into separate tubes and immediately frozen also at -80°C. Thus, the tissue components were stored at the time of their surgical collection over a 12–18 month period through until a single thawing for subsequent laboratory analysis.

Peripheral blood samples were collected from LE patients using both SST-II and K_2_EDTA vacutainer collection tubes immediately prior to anaesthesia for liposuction surgery. Peripheral blood was also obtained from non-related healthy age and sex matched donors (n = 13; Table 1) by standard venepuncture of peripheral veins, performed by trained phlebotomists. All LE patients and healthy donors they were “fasting” and “resting”; they consumed no food and did not exercise for at least 8–10 hrs prior to venepuncture or AT collection by liposuction surgery. Peripheral blood samples were centrifuged for 10 minutes and the serum (and plasma) layers removed, aliquoted, then stored frozen until a single thawing for laboratory analysis.

### Automated biochemical analysis for total serum lipids

Total serum lipids were measured as follows: cholesterol was measured with a cholesterol enzymatic assay (Abbott), triglycerides with a glycerol phosphatase oxidase based assay (Abbott), and HDL-Cholesterol with the Ultra HDL accelerator selective detergent assay (Abbot) and analyzed on a Abbott Architect c16000 instrument at Douglas Hanly Moir Pathology, Macquarie Park, Sydney. The LDL-cholesterol levels were calculated as follows: LDL-cholesterol = cholesterol–HDL-(0.45 x triglycerides), which is essentially similar to both the Friedewald and Amhadi formuli [24,25]. The apolipoprotein-A1 and -B serum levels were determined by immunoturbidimetric assays Tina-Quant Apolipoprotein A-1 Ver 2 and Apolipoproteins B Ver 2 (Roche), using the Roche Integra 800 analyser at the Sullivan and Nicolaides Laboratory in Brisbane, Australia. The generally accepted normal range of these molecules in human serum is noted together with the data.

### Gas Chromatography—Mass Spectrometry (GC-MS) fatty acid analysis

AT samples of approximately 50 mg were extracted into 1mL chloroform:methanol (2:1 v/v) by incubation on a thermomixer (Eppendorf) for 10 min at 30°C, then centrifuged at 18000g for 5 min at 4°C. A 50 ul aliquot of the supernatant from each sample was also combined to create a pooled biological quality control (PBQC). Individual samples and PBQC (5 ul) were transferred to a glass insert to which 40 ul of internal standards were added: chloroform:methanol (2:1 v/v) containing 62.5 uM each of ^13^C_18_-stearate and ^13^C_2_-myristate. Straight chain fatty acid (Sigma) standards (25 uM) and monomethyl branched-chain and cyclopropane-type fatty acid standards (25 uM) were synthesized in-house as described previously [26,27] and prepared in chloroform:methanol (2:1). Inserts containing AT samples and the PBQC were sealed into GC vials and transesterified to create fatty acid methyl esters. For this samples were mixed with 5 uL Methprep-II (Alltech) using a Gerstel MPS2 autosampler robot, incubated for 30 min at 37°C with slow shaking for mixing. Fatty acid analysis was performed on an Agilent 7890 gas chromatograph coupled to a 5975 mass selective detector. Samples (2 ul) were injected in split mode (10:1) into an inlet set at 250°C and chromatographic separation was achieved on a SGE BPX70 column (60 m x 0.25 mm i.d. x 0.25 um film thickness). The oven conditions were 70°C for 1 min, then 40°C/minute to 150°C, 4°C/minute to 200°C, 3°C/minute to 220°C, 4°C/min to 250°C then held at 250°C for 4 minutes. Helium carrier gas flow was constant at 1.5 ml/min and the MS transfer line was set at 280°C. Compounds were ionized using electron impact fragmentation (-70eV) and mass spectra collected over the 50–450 m/z range at 3.6 scans/second.

### Liquid Chromatography-Mass Spectrometry (LC-MS) lipid analysis

LC-MS lipid analysis was performed essentially according to standard methods described previously for blood lipids [28]. Briefly, fat-oil (~50 mg “Oil”) and solid-fat (~50 mg “Fat”) samples were extracted using 1 mL chloroform:methanol (2:1, v/v) containing the internal standard 5 uM C17:0 LPC for 1 hr at RT. The extracts were centrifuged at 18000 g for 10 minutes at 4°C (in a refrigerated microcentrifuge) and the supernatants transferred to a new tube. The pellet was re-extracted with 500 ul chloroform:methanol (2:1, v/v), centrifuged as described above and the supernatants combined. The fat-oil and solid-fat extracts were dried under nitrogen gas then resuspended in 100 ul butanol:methanol (1:1) containing 5 mM ammonium formate (Sigma). Lipids were analyzed using an Agilent 1200 liquid chromatography (LC) system and Triple Quadrupole 6460 mass spectrometer (MS). Briefly, lipids were separated by injecting 5 ul of the sample onto a 50 mm × 2.1 mm × 2.7 μm Ascentis Express RP-Amide column (Supelco) followed by elution at flow rate of 0.2 ml/min over a 5 min gradient of water/methanol/tetrahydrofuran (50:20:30, v/v/v) to water/methanol/tetrahydrofuran (5:20:75, v/v/v) with the final buffer held for 3 min. Lipids were identified by electrospray ionization-mass spectrometry using a method that was optimized using a panel of authentic lipid standards. Lipid identification was carried out using two scan functions available in triple-quadruple mass-spectrometer, a precursor scan and neutral loss scan. Both scans are profiling identification of precursor masses of the lipid species based on the m/z of the fragment of the head group. Phosphatidylcholines (PC) and sphingomyelins (SM) were identified by scanning for precursors at 184.1 m/z, whilst ceramides (Cer), cholesterol esters (CE) and phosphatidylglycerols (PG) were identified by scanning for precursors at 264.6, 369.4 and 189 m/z respectively, all in positive MS mode. Phosphatidylethanolamines (PE) were identified by scanning for neutral loss 141 m/z in positive MS mode. Diacylglycerol (DAG) and triacylglycerol (TAG) were identified using neutral loss scans of possible fatty acyl moieties. Thus the lipid identifications are putative and most have not been validated by other means. Lipids were quantified by using multiple reaction monitoring with capillary voltage 4000V, fragmentor voltage 140–380 V, collision energy 15–60 V and collision gas (nitrogen) at 7 L/min. Quantitation was based on relative changes in peak areas.

### Data handling and statistical analysis

Straight-chain lipid species are uniformly refered to via the Cx:y nomenclature which refers to the total number of carbons (x) and number of double bonds (y) in the lipid. For example, cholesterol ester CE(14:1) has 14 carbons and 1 double bond—note that the position of the double bond is not defined. Branched chain fatty acids studies include iso-fatty acidsm containing a methyl group at the penultimate carbon. Thus, iso-C15:0 refers to 13-methylmyristic acid. Cyclopropane fatty acids studied include cis-9,10-methylenehexadecanoic acid (MHA), dihyrosterculic acid (DHS) and lactobacillic acid (LBA). The lipid nomenclature and classification conforms to that in LIPD MAPS http://www.lipids.org. Both GC- and LC-MS data was processed using Agilent Mass Hunter Quantitative Software (B.06.00). Raw data indicating relative abundance (area under curve) was analyzed in Microsoft Excel (Version 14.5.9 for Mac OS), calculating mean, standard deviation (SD) and median for LE or normal cohort data. Raw data was first log transformed, then adjusted by median normalization (x/median) [29] where the median value as individually determined for each class of lipid included in the study (e.g. fatty acids, DAG, TAG, CE, PE/LPE, SM, Cer, etc), in order to account for the heteroscedastic nature of metabolomics data. Where a lipid was undetected a zero value was replaced by “0” or “1” to permit downstream analysis (raw data, boxed cells in supplementary data). Means and SD were again calculated this time from the log transformed median-normalized data, and significant differences between LE versus normal AT samples, assessed each for lipid species, identified using an two-tailed unpaired Student’s t-test; statistical significance p<0.05, with no assumptions for data distribution (normality). Global-median normalized data were also independently analyzed to permit a principle component analysis (PCA) that assessed the global differences between all metabolites within each of the sample cohorts simultaneously; the PCAs were performed using R software and the results can be found in S1 Fig. The data were additionally examined to identify potential false discoveries using the Benjamini-Hochberg correction method [30]. Finally, the data was analyzed for correlation to LE years by the Pearson correlation method using Graphpad PRISM (version 60f for Mac OS X), with the corresponding R^2^ values tabulated together with *p* < 0.05 as an indicator of significance. The data were graphed with PRISM and multicomponent data figures were constructed in Canvas Draw 2 (version 1.01) for Mac OS.

## Results

### Total serum lipids in advanced LE

**Fig 2:**
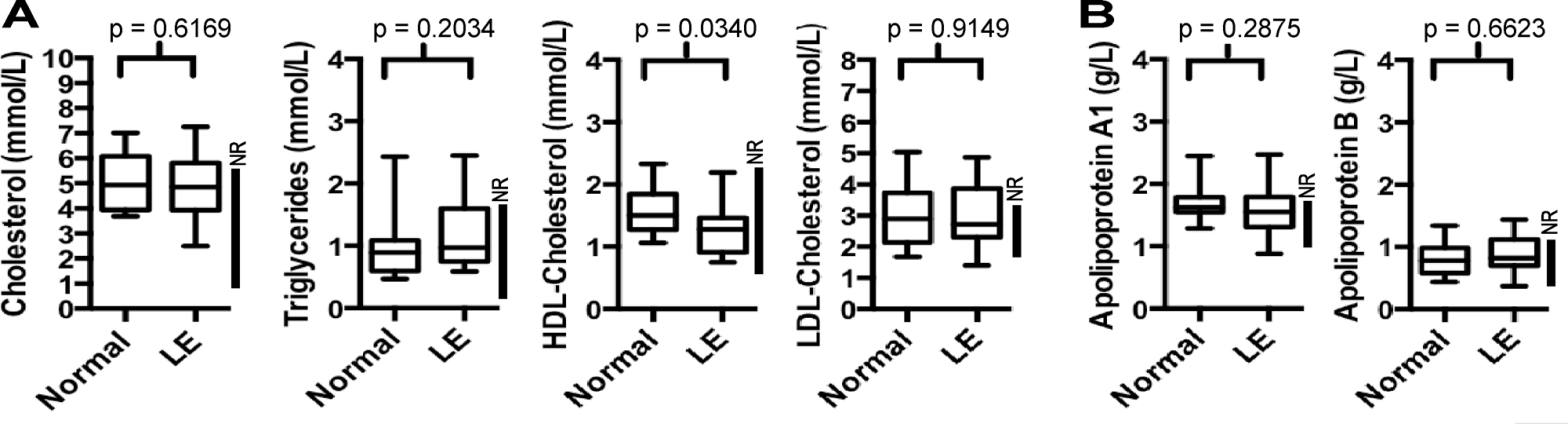
Total Serum Lipids and Lipid Transport Molecules. (A) Box and whisker plots of total serum cholesterol, triglycerides, HDL-cholesterol and calculated LDL-cholesterol, and (B) Total serum lipoprotein apoprotein A1 and apolipoprotein B, from LE patient (n = 15) and normal cohort (n = 11) peripheral blood samples. The line within each box indicates the global median value calculated from the cohort. The generally accepted population normal range (NR) is shown within each graph (vertical line, right hand side). Statistical significance (*p* value) between LE and Normal (non-LE) are also shown, as determined by two-tailed t-test, not assuming equal variance.

To determine whether there was any systemic lipid-based metabolic imbalance associated with limb AT deposition in chronic advanced cancer-related LE, 15 LE patient serums (Table 1) were analyzed for total circulating cholesterol and triglycerides levels, and compared 13 normal healthy sex- matched serum from healthy donors. The serum levels of these molecules were highly similar in LE patients as was present in healthy, non-LE, blood donors, and both cohorts contained several hypercholesterolemic individuals i.e. individuals with cholesterol levels above the recommended levels for healthy individuals, i.e. above the “normal range” (Fig 2A). Despite these similarities, the serum HDL-cholesterol levels were found to be lower (statistically significant, p = 0.0340) in LE patients compared to the healthy donor cohort samples, albeit these samples were still within the normal range (Fig 2A). The calculated LDL-cholesterol levels were also similar between the LE and normal cohort samples (Fig 2A), as were the serum lipid transport molecules, apolipoprotein A1 and apolipoprotein B (Fig 2B). Thus, the abundance of total serum lipids and lipid-transport molecules are largely unaltered in advanced cancer-related LE compared to normal donors, and within the normal range for healthy adults.

### Serum lipidomic analysis of LE Patients

Because these rapid diagnostic routine laboratory assays do not provide information on the type or abundance of individual lipids species, 7 randomly-chosen LE patient samples and 3 “normal” (healthy) donor serum samples were analyzed further by gas chromatography mass spectrometry (GC-MS). This permitted the specific molecular identification of some 31 fatty acids in human serum, including 4 branched-chain fatty acids, the latter not frequently analyzed in other publications investigating serum lipids. Nevertheless, and consistent with the total serum lipid levels, no individual serum fatty acids were found to be present in LE patient serum with an altered abundance than was present in healthy “control” (non-LE) cohort serum (data now shown; S1 Data File; Sheet “Serum”). A principle component analysis (PCA) was also performed, but, as expected, no significant global differences were evident between the LE and normal serum samples, as all the LE and normal cohort data points were broadly inter-dispersed (data not shown). Taken together with the total lipid serum analysis described above, these data indicate that the presence of large amounts of AT within the LE-affected limbs of advanced cancer-related limb LE patients does not result in detectable differences in the profiles or levels of circulating blood-born lipids, as assessed by these methods. Moreover, this result aligns closely with the normal abundance of serum transport proteins apolipoprotein A1 and B in LE serum (referred to above, Fig 2).

### Lymphedema adipose tissue (AT) lipidomic analysis

Despite the lack of difference in total serum lipids, it remained unknown if the LE AT itself consisted of unusual lipid molecule profile, or an altered relative abundance compared to normal human limb AT. We therefore molecularly analyzed both the solid AT (“fat”) comprising intact adipocyte-rich tissue, as well as the already released adipocyte oil (i.e. fat oil; referred to hereafter as “oil”) present in lipoaspirates obtained from LE patients undergoing liposuction surgery, and compared this to the fat and fat oil present in the lipoaspirates obtained from healthy individuals undergoing liposurgery for cosmetic reasons. Of note, all lipoaspirate samples were anatomically-matched, i.e. all were arm or leg AT and both the solid AT fat, and fat oil, were analyzed separately by GC-MS and LC-MS. This permitted the simultaneous identification and relative quantification (log transformed median-normalized relative abundance) of some 275 lipid-based molecules, including: 25 diacylglycerides, 66 triacylglycerides, 34 fatty acids, and 150 phospholipids of various classes, in fat and fat oil in human lipoaspirate tissue samples.

**Fig 3:**
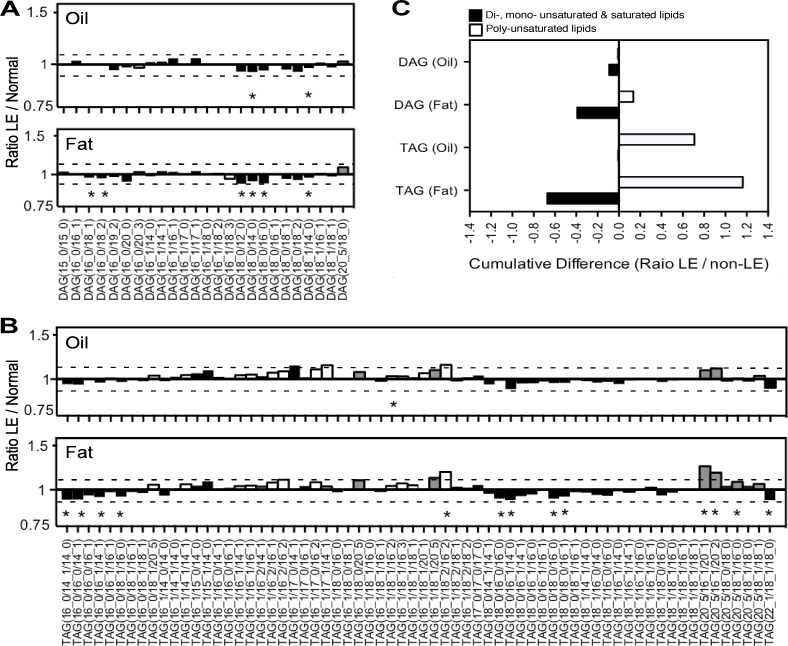
AT Diacylgleycerides (DAGs) and Triacyglycerides (TAGs). (A) DAG lipids and (B) TAG lipids in LE AT versus cosmetic surgery AT lipoaspirate fat (Fat) or the liquid fat oil (Oil). Data shown are ratios of LE compared to Normal non-LE AT, calculated from means ± SD of log transformed median-normalised relative abundance. Dotted line indicates ± 0.1-fold difference; Poly-unsaturated lipds with ≥ 3 unsaturated bonds (white fill) or containing C20:5-based lipids (grey fill), compared to saturated, mono- or di- saturated lipids (back fill). Statistical significance as noted (*), *p* < 0.05, based on two-tailed unpaired students t-test, irrespective of whether the difference is ±0.1 with respect to normal AT. (C) Total number of poly-unsaturated versus combined unsaturated plus mono- or di- unsaturated lipids in LE and non-LE oil and fat AT.

First, we used LC-MS to identify diacylglycerides (DAG) and triacylglyceride (TAG) lipids in both LE and normal limb AT. Although most of the DAGs and TAGs that were identified in oil and fat of human LE AT were present in similar abundance as that detected in normal limb AT (i.e. between -0.1 to +0.1 reduced or increased comparing LE to normal), there were several lipid species that were individually statistically significantly increased or reduced in LE compared to healthy tissue (Fig 3A and 3B, and S2 Data File). While these changes were each quite small, it appeared that most of the increases were in TAGs with polyunsaturated fatty acids, and notably those containing 3 or more unsaturated carbons (white bars), especially eicosapentaenoic acid C20:5 (grey bars), both in LE AT fat and oil (Fig 3A and 3B). Moreover, when these data were combined together it appeared that there may be a global increase in poly-unsaturated fatty acid containing TAGs and a concomitant reduction in less-saturated fats, such as di-, mono-, or completely unsaturated fats, in LE AT (Fig 3C). However, in mammals, C20 poly-unsaturated fatty acids are generally synthesized from dietary sourced linoleic acid (C18:2) and alpha-linenic acid (C18:3) through the action of fatty acid desaturases and elongases, and the proximal enzyme producing both arachidonic acid C20:4 and eicosapentaenoic acid C20:5, from their respective precursors C18:2 and C18:3, respectively, is the delta-5-desaturase. Conversely, mono-saturated fatty acids are reliant on stearoyl-CoA desaturases. Thus, it remains unclear whether these small individual changes in specific DAG and TAGs are biologically significant i.e. whether (i) they reflect real changes in lipid metabolism such as an increase in delta-5-desaturases and/or reduced stearoyl-CoA desaturase activity, that subsequently or concomitantly result in increased production of the anti-inflammatory omega-3-based eicosapentaenoic acid C20:5, or alternatively (ii) whether these small effects are all simply within realms of background variation—especially given their small magnitude and general lack of statistical significance after BH correction analysis. Nevertheless, a reasonable initial conclusion is that the LE AT appears to have a remarkably similar lipidomic profile to non-LE AT (except for the higher content of C20:5 containing TAGs), and that the LE oil that is liberated from ruptured adipocytes resembles the oil that is present within intact AT adipocytes (fat)—at least with respect to the DAG and TAG content. This conclusion was further supported by globally-normalized data principle component analysis (PCA) considering all 66 DAG and TAGs, since LE fat and oil data points were modestly separated away from non-LE samples (S1 Fig). This conclusion is also supported by the fact that none of the statistically significant DAGs and TAGs were positively correlated to LE years (data not shown).

**Fig 4:**
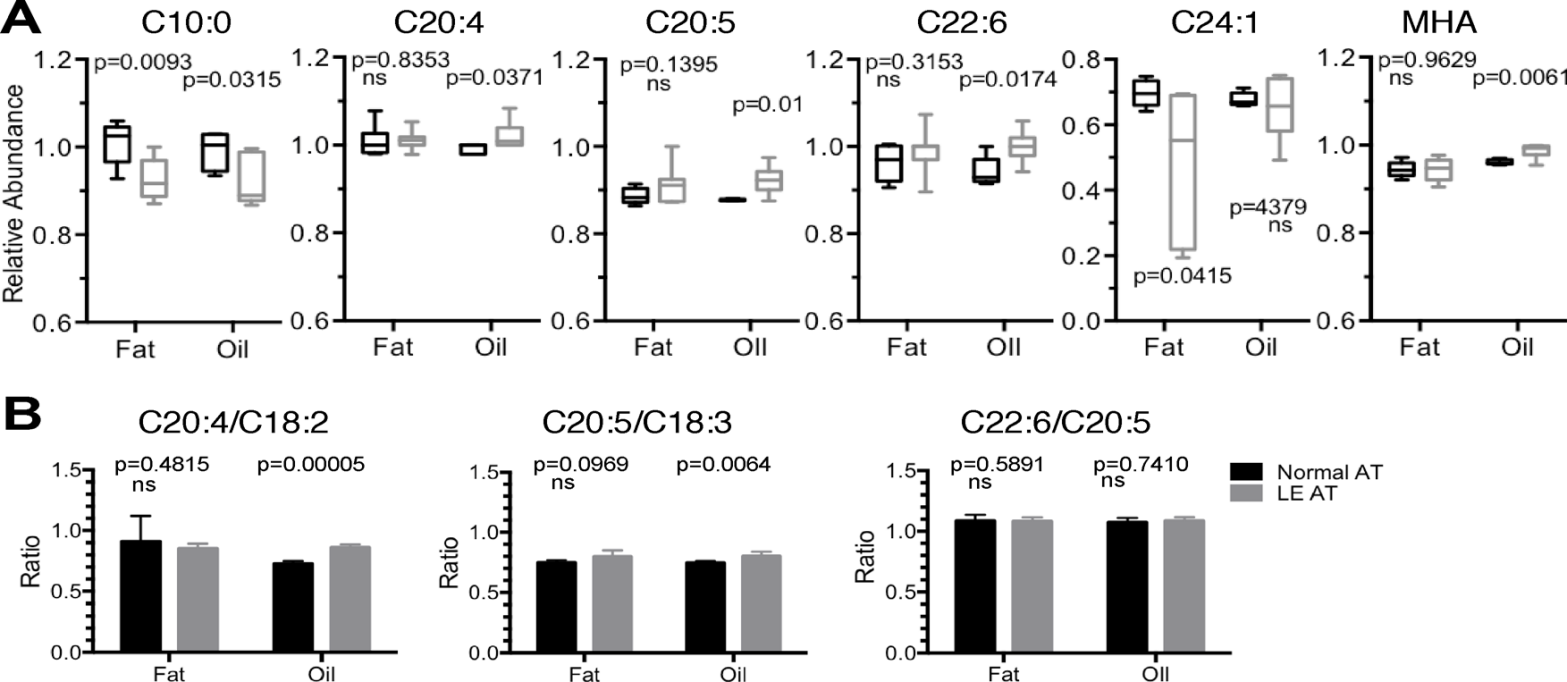
AT Fatty Acids. (A) Box and whisker plots of relative abundance (median-normalised log transformed data) of individual fatty acid species found to be statistically significantly altered and > ± 0.1-fold altered in LE patient AT fat or fat oil (grey) compared to normal AT fat or fat oil (black) (Lipid species referred to with Cx:y namenclature to define the total number of carbons in the fatty acid chain and double bonds). (B) Ratio of C20:4 arachidonic acid and C20:5 eicosapentaenoic acid relative to their precursors, C18:2 and C18:3 linolenic acids, respectively. Statistical significance as noted, determined by two-tailed students t-test with *p* < 0.05 not assuming equal variance, or no significant difference (ns) detected.

Next, GC-MS was used to identify some 34 individual AT fatty acids. As with the earlier analysis on serum, the majority (28 of 33) of the identified fatty acids were found to be present in similar levels in LE as compared to that found in normal (non-LE) AT, and furthermore, the similarities were evident even when analyzing AT fat independently to the AT oil (see S1 Data File, sheets “Oil” and “Fat”). However, this same analysis also indicated that there were in fact some important differences: 6 of the 34 identified fatty acids were statistically altered in abundance in LE AT compared to non-LE AT. First, C10:0, otherwise known as decanoic acid (capric acid), was present in reduced amounts in LE AT fat and oil compared to non-LE AT (Fig 4A). Second, C20:4 arachidonic acid, C20:5 eicosapentaenoic acid (EPA), and C22:6 docosahexaenoic acid were individually statistically significantly elevated in LE AT oil compared to normal non-LE AT oil, but not in LE AT fat (Fig 4A, see S2 Data File). Also evident, and strikingly so, was a broad reduction of the long-chain monounsaturated fatty acid C24:1 nervonic acid in LE AT fat compared to normal non-LE AT fat, even though the effect was only evident in AT fat but not oil (Fig 4A). Of note, several branched chain and cyclopropane fatty acids, such as 9,10-methylene hexadecanoic acid (MHA), were found at detectable levels in human AT, and furthermore, MHA was statistically significantly increased in abundance, but only in LE AT oil compared to non-LE AT oil, and not in fat (Fig 4A, S2 Data File). Given that the change in MHA abundance is only very small, and statistically significant in a standard t-test but not significant after BH adjustment, the biological significance of this finding is also currently unclear. Of interest, however, the increase in broadly anti-inflammatory omega-3 fatty acids eicosapentaenoic acid C20:5 and docosahexaenoic acid C22:6, both noted in the individual analysis, significantly positively correlated with years of LE, unlike the other differentially abundant lipids (Table 2). As with the earlier analyses, a principle component analysis was performed. Consistent with the specifically affected lipids, mentioned above, the PCA analysis indicated that the LE AT fat and oil were to some extent separated from normal (non-LE) fat and oil, albeit with a broad degree of data spread—probably reflecting sample heterogeneity (S1 Fig).

**Table 2:** Correlation analysis of altered AT fatty acids and phospholipid abundance with LE years.

LE Years correlated^a^ with:	C10:0	C20:4	C20:5	C22:6	C24:1	MHA	CE(14:0)	CE(16:0)	CE(17:2)	CE(22:5)	PC(33:4)	PC(32:6)	Cer(d18:1/28:3)	HexCer(d18:0/23:0)
**Oil**														
R^2^ p	0.1358 0.4159	0.0128 0.8146	0.5306 0.0634	0.5780 0.0473	0.0706 0.5647	0.0764 0.5486	0.4797 0.1272	0.0063 0.8809	0.2731 0.2874	0.0619 0.6345	0.1698 0.4169	N/a N/a	0.0712 0.6094	0.1052 0.5305
Significant*^b^*:	No	No	No	Yes	No	No	No	No	No	No	No	-	No	No
**Fat**														
R^2^ p	0.0153 0.7916	0.3185 0.1869	0.7057 0.0180	0.6430 0.0301	0.1736 0.3525	0.2822 0.2198	0.1239 0.5612	0.0156 0.8414	0.2882 0.3509	0.0703 0.6663	0.1564 0.5099	0.0001 0.9840	0.0344 0.7652	0.0486 0.7217
Significant*^b^*:	No	No	Yes	Yes	No	No	No	No	No	No	No	No	No	No

^*a*^ Pearson correlation calculation, compares LE years of individual samples to log transformed median-normalized lipid species found to be statistically significantly altered in abundance in LE AT compared to non-LE AT. R^2^ is degree of alignment to line of best fit.

^*b*^ Significance equal to p <0.05; p value (two-tailed). N/a; Not applicable–not detected i.e. not present in AT oil (<0.001 units).

It is well known that C20:4 arachidonic acids originate from both lipid metabolism from its precursor linoleic acid C18:2, via the actions of elongation and desaturation enzymes, and originally from dietary sources [31]. Similarly, C20:5 eicosapentaenoic acid is generally produced from the precursor lipid C18:3. Thus, since arachidonic acid is an important precursor of pro-inflammatory intermediates eicosanoids and ceramides we therefore further assessed the data by determining the ratio of C20:4 and C20:5 to their precursor lipids C18:2 and C18:3, as described previously in lipid biosynthesis studies [32,33]. Consistent with the above results, LE AT oil contained statistically significantly increased ratios of C20:4 and C20:5 compared to precursor molecules, but there was no significant difference in C22:6/C20:5 ratio, either in AT oil or fat (Fig 4B). These combined data, taken together, can be reasonably taken to indicate that (i) fatty acid metabolism is essentially normal in LE AT, (ii) there is a reduced presence of caprinic and nervonic acids–currently of unknown biological significance, and (iii) specific increases in pro-inflammatory lipids such as arachidonic acid C20:4, subsequent to or concomitant with increased anti-inflammatory omega-3 eicosapentaenoic acid C20:5 and docosahexaenoic acid C22:6. Because arachidonic acid is a precursor to the production of prostaglandins and since LE is a life-long chronic clinical condition, a reasonable interpretation of this data is thus that LE may henceforth represent a condition of chronic low-grade inflammation, and the generally anti-inflammatory actions of C20:5 and C22:6 may constitute a compensatory increase in bio-synthesis and/or physiological accumulation.

**Fig 5:**
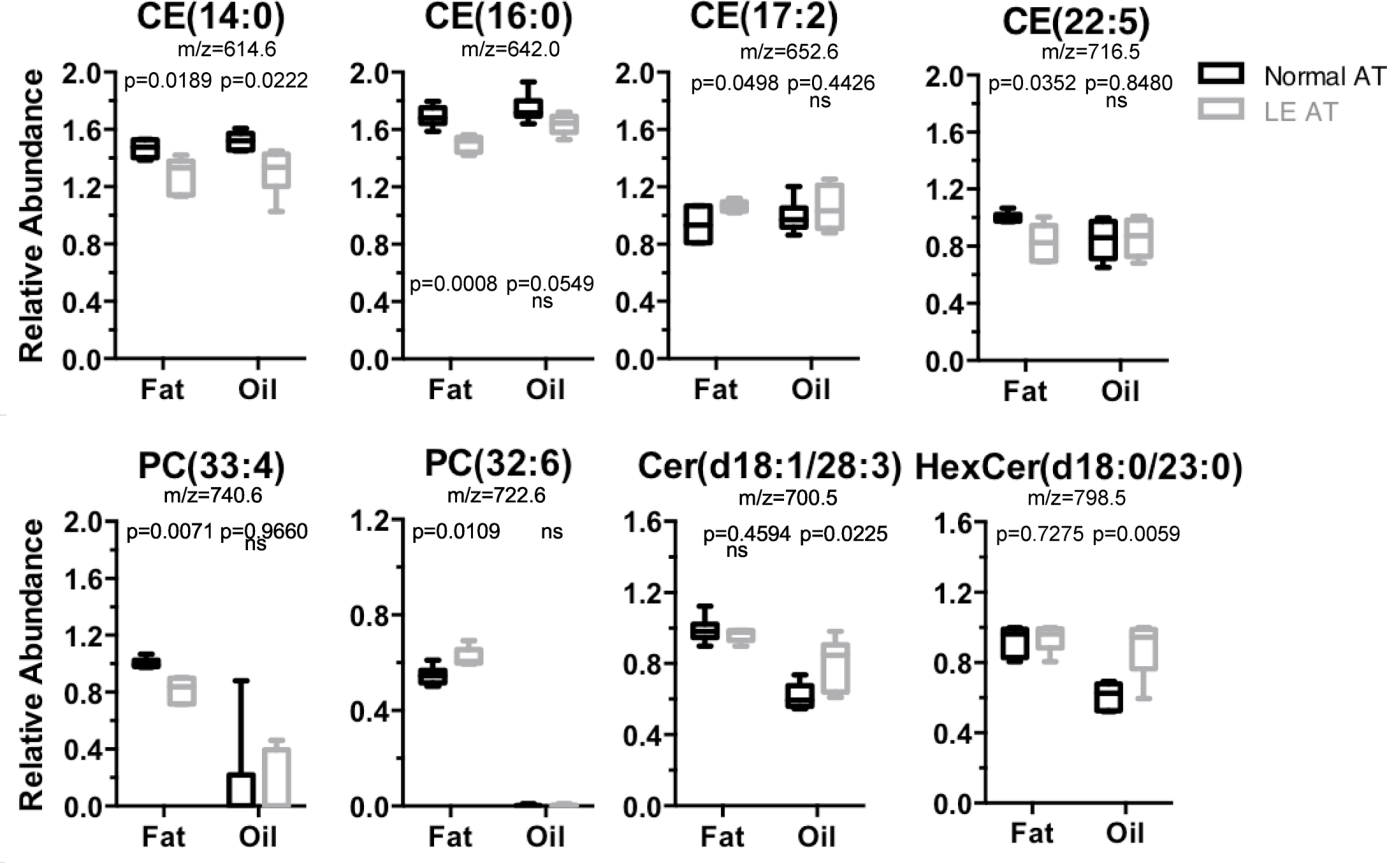
AT Phospholipids. Box and whisker plots of relative abundance (median-normalised log transformed data) of individual lipid species for the major phospholipid types: cholesterol esters (CE), phosphotidylcholines (PC) and ceramindes (Cer, including hexaceramides HexCer). m/z values are also shown, note that CE species detected as ammonium adducts (NH_4_^+^) while PC and Cer are detected as protonated species (H^+^); See S3 Data File for a full list of all phospholipid species detected. Statistical significance as noted, determined by two-tailed students t-test with *p* < 0.05 not assuming equal variance, or no significant difference (ns) detected.

To complete the lipidomic analysis of LE AT, LC-MS was used to investigate the presence and relative abundance of phospholipids–these molecules being the major types of lipids in cellular membranes. This permitted the identification of 133 phospholipid-based molecules, including a broad array of lipid species comprising 51 phosphatidylcholines (PC; including 6 lyso-phophatidylcholines, LPC), 47 phosphatidylethanolamines (PE) (including 4 lyso-phosphatidylethanolamines, LPE), 18 cholesterol esters (CE), as well as 25 ceramide (Cer) and hexa-ceramides (HexCer), and 19 sphingomyelin (SM) type lipids. In both the LE AT samples and the normal (non-LE) AT samples, the majority of the phospholipid molecules were found to be present in highly similar relative abundance, even when analyzing LE AT fat or AT oil separately (S3 Data File). Furthermore, the PCA demonstrated distinct clustering of LE and normal (no-LE) fat i.e. away from LE and non-LE AT oil (S1 Fig). This is consistent with the fact that phospholipids are normally components of membranes rather than free oil. Indeed, even when statistically significant individual lipid differences were detected, it was apparent that the fold-difference from healthy (non-LE) AT, were usually extremely small i.e. between -0.9 and 1.1, and these small changes were generally considered to be potentially biologically non-significant. There were, however, certain phospholipids that were both statistically significant (p<0.05) and comprising of a magnitude greater than 0.1-fold changed with respect to the normal AT—as follows: cholesterol esters CE(14:0) and CE(16:0) were decreased by a factor of 0.87 and 0.88, and CE(22:5) was decreased 0.82, in LE AT compared to non-LE AT, whereas CE(17:2) was elevated approximately 1.13-fold (Fig 4). A single phosphatidylcholine, PC(33:4), was decreased by a factor 0.811, while PC(32:6) was increased by 1.13, compared to non-LE AT (Fig 5 and S3 Data File), and there were no discernable changes in any of the ceramides and sphingomyelin lipids detected in LE AT fat. However, and in contrast to the AT fat, an analysis of AT oil revealed the following significant differences (greater than 0.1-fold from non-LE AT oil): CE(14:0) was 0.85-fold reduced (similar to that found in LE AT fat) but there were no other changes in other CEs in LE AT oil (Fig 5 and S3 Data File). On the other hand, it was clear that some 11 of 25 detected ceramide species were statistically altered in LE AT oil and even though many were small changes (approximately ≤0.1-fold), with the most striking result within the entire LC-MS phospholipid analysis, even within the entire lipidome analysis, being the significant elevation of Cer(d18:1/28:3) by 1.30-fold and HexCer(d18:1/23:0) by approximately 1.44-fold in LE AT oil compared to non-LE AT oil (Fig 4, and S3 Data File). (Note: the mass/charge values are indicative of the putative ceramide identities). Lastly, while it was evident that there were some significant reductions in a number of the PC-type lipids, such as PC(32:0), PC(33:1), PC(34:0), PC(34:1), PC(34:2), PC(35:4), PC(36:1), PC(36:2) and PC(36:4) in LE compared to non-LE AT oil, these molecules are usually present in membranes rather than in free AT oil. This result appears most likely to be artificially exaggerated by contamination effects likely due to the imprecise, and difficult, complete separation of AT fat from AT oil. In considering the overall results of the phospholipid analysis, the biological significance of CE(14:0) and CE(22:5) being reduced in LE AT, is currently unknown, while the increase in ceramides are usually considered an hallmark of inflammation [34]. Moreover, the presence of both increased arachidonic acids (mentioned above) and increased ceramides in LE AT oil, rather than the intact adipocytes, was particularly notable and may indicate that an inflammatory pathology is active within LE tissue. Of note, none of these altered phospholipids were correlated with LE years (Table 2) probably indicating that the inflammation is a constituent with components inherently residing within the AT.

In summary, these results of this lipidomic analyses of LE lipoaspirates demonstrates that the AT associated with advanced limb LE contains many lipid-based molecules that are normally present in healthy, non-LE, anatomically-matched, human limb AT. That the similarities generally extended across the full spectrum of lipid-based molecules including PC, SM, Cer, CE, PG and PE phospholipids, diacylglycerides and triacylglycerides, as well as fatty acids, again indicates that LE adipocytes are themselves, for the most part, essentially the same as normal (non-LE) AT adipocytes i.e., that lipid metabolism in LE AT is globally similar to that which occurs in normal (healthy) limb AT. Nevertheless, given that LE is a chronic condition, indeed often life-long in cases of primary LE, and that we document a distinct lipid pro-inflammatory signature comprising arachidonic acid and ceramide-type lipids in LE AT oil, suggests that an undefined pathological and pro-inflammatory process is active LE AT. Whether chronic low-grade or localized acute inflammation causes the liberation of the free oil that frequently appears in LE tissue lipoaspirates (Fig 1C), or whether the free AT oil is simply a consequence of constant massage-based treatments and compression (to minimize LE swelling) but inherently capable of provoking an endogenous inflammatory signature, remains to be determined. Nevertheless, since LE years correlates positively with the accumulation of generally protective omega-3-type C20:5 and C22:6 lipids, it appears that this occurs in response to the chronic low-grade inflammation that is inherent within the LE AT.

## Discussion

This study examined lipoaspirates from patients with primary and cancer-related limb LE, comparing the tissue to that obtained from patients undergoing cosmetic liposuction surgery; it documents many similarities as well as specific small differences in lipidomic profiles between LE and non-LE AT. The study was carefully controlled from a number of perspectives. First, all the tissue donors, including both LE, cosmetic surgery patients, and normal tissue donors, had a calculated BMI of less than 30. The only exceptions were 2 LE patients for whom the BMI ratings at the time of surgery were greater than 30, but this appeared to reflect their enlarged LE-affected limbs rather than a state of obesity. Thus, all study participants were categorized as either “normal” or “healthy, and overweight” but not obese. Second, the tissue samples were all limb (arm or leg) AT, rather than abdominal or visceral AT and all AT samples were obtained by via liposuction surgery. This additionally meant that all of the AT donors were both fasting and resting—a requirement that was also matched by the normal blood donors. This is important because human blood (plasma) lipid profiles can be influenced by diet and exercise [35,36]. Third, there are several considerations specific to the surgical procedure itself. Most liposuction protocols involve the post-surgical administration of a saline-based adrenalin infusion to limit hemorrhage. This study, however, examined only lipoaspirate tissue collected prior to the administration of adrenaline. Furthermore, cosmetic liposuction surgery usually involves the use of saline in the tissue extraction procedure, but no additional fluid is required for LE liposuction (due to the presence of accumulated lymphatic fluid within the swollen LE tissue). Thus differences in surgical procedures should have little or no influence on the data generated in this study because normal saline will not alter the lipid constituents. This study therefore represents an important and rare opportunity to undertake a comprehensive molecular analysis of human LE AT. In fact, this study appears to provide the first publicly available data of the lipidomic analysis of cancer-related and primary LE AT. Nevertheless, there are two potential confounders that are difficult to content with in a lab-based analysis of this nature. One is simply the scale of the tissue samples that are available from liposuction surgery compared to the small amount of tissue that is required for these types of analyses. For example, liposuction surgery can yield several liters of lipoaspirate tissue whereas only 50 mg of tissue sample is required for the MS analysis. The inherent heterogeneity within the sample is therefore only addressed by way of the number of samples that are examined. The other potential confounder is the statistically significant age difference between the LE AT cohort (mean age 49) and the healthy (non-LE) AT cohort (mean age 26; see Table 1). This was unavoidable due to the limited availability of normal human adult AT collected by liposuction surgery and, more importantly, it may be biologically insignificant as it is unknown if there are major changes in AT in healthy adults within the 20–60 year age bracket. The comparison of these AT sample cohorts, despite being statistically significantly different in age, was therefore considered to be valid, but this difference highlights the need for more normal but age- and gender- specific lipidomic and proteomic data to be published or available in open access repositories. Therefore, while this study represents the first substantial lipidomic analysis of LE AT, subsequent independent studies will be required to validate our findings and to fully complete the lipidomic profile of advanced human LE AT.

Since mammalian tissues produce diverse lipids through normal metabolism and excrete them, thus, the serum lipidome is considered to reflect the recent history of tissue lipid metabolism within an individual. Circulating lipid profiles are therefore thought to provide a signature indicative of abnormal tissue metabolism. While this is true in diverse disease states [37], it is especially valid in conditions associated with significant changes in tissue adiposity such as obesity [38] or non-alcoholic steatohepatitis (NASH)[39]. Of note, these are conditions that are both characterized by abnormally increased tissue adiposity and subsequently also with fibrosis [40,41]—similar to what that in human LE tissue and mouse models of LE [42–45]. The lack of an altered serum lipidomic profile in LE patients with extreme limb-localized adiposity, i.e., in advanced LE, was therefore, arguably perhaps quite unexpected. On the other hand, our results are consistent with a previous study, albeit a considerably more limited analysis comprising only of total serum lipids in LE patients [46]. Furthermore, it may be important to consider that obesity and NASH are both conditions involving visceral and organ adiposity whereas these LE patients experience only localized peripheral limb adiposity, and, moreover, all the LE patients recorded normal BMI ratings. Our data therefore suggest that LE is not itself a condition of fundamentally altered or grossly defective lipid metabolism. Moreover, the findings presented here can be interpreted to indicate that reverse cholesterol transport is only minimally, and perhaps not biologically significantly impacted, in advanced human cancer-related LE; while we acknowledge that serum HDL cholesterol was statistically slightly decreased, it was still within the normal range and neither total serum lipids nor serum lipoproteins were unchanged from normal, in LE patients. It appears instead that the development of LE AT may simply be directly due to the accumulation of lymphatic fluid which has long been known to be a lipid-rich substance [47–49]; cholesterol-HDL-rich lymphatic fluid stasis therefore provides a perfect environment of active lipid metabolism capable of supporting both nascent adipocyte development and adipocyte hypertrophy, as reportedly occurs in murine models of lymphedema [50].

Despite the overwhelming global similarities between LE and non-LE AT, there were certain intriguing lipid differences between LE AT and non-LE AT. One was the decrease in caprinic acid C10:0 in LE at fat and oil, and another was the broad and significant reduction in the long-chain mono-unsaturated fatty acid nervonic acid C24:1 in LE fat. Little is known about the role of caprinic acid in human AT and although it has been suggested to have potential antimicrobial properties [51], whether its reduced presence in LE AT fat and oil represents diminished production or increased catabolism, is unknown. Furthermore, even less is known about nervonic acid in human AT as it is predominantly found in brain where it normally comprises up to 40% of sphingolipids [52]. Curiously, recent reports suggest that decreased erythrocyte nervonic acid correlates with psychosis in ultra-high risk individuals [53], and in an unrelated study, nervonic acid has been reported to be reduced in the umbilical chords in women with preeclampsia, and for unknown reasons, specifically in women who deliver male babies [54]. Thus, the biological significance of reduced nervonic acid in LE AT, like caprinic acid, are currently unknown. Nevertheless, it is tempting to speculate as to the biological significance of these findings: the decrease in nervonic acid might suggest adipocyte peroxisome and mitochondrial stress causing long chain fatty acid oxidation because very long-chain fatty acids generally undergo oxidation within peroxisomes and mitochondria. In peroxisome oxidation, FADH_2_ generates hydrogen peroxide (H_2_O_2_), and hence the outcome of sustained or over-active peroxisomal oxidation of long-chain fatty acids would correlate with a phenotype of stressed adipocytes and, perhaps, even ultimately in adipocyte death—depending on the levels of H_2_O_2_. Alternatively, as with caprinic acid, its decrease might reflect either a decrease in production, or its increased utilization i.e. catabolism, and further research will be needed to determine which. Thus there is little that can at inferred with any confidence, at present, about roles of caprinic and nervonic acid in human AT.

Another of the most obvious results of this study was the elevated relative abundance of poly-unsaturated TAGs, especially those containing C20:5, which occurred concomitant with the increase in individual fatty acids C20:4, C20:5 and C22:6. Because the C20:4/C18:2 and C20:5/C18:3 ratios were only slightly increased in LE AT oil (but not in AT fat), and since the C22:6/C20:5 ratio was unaltered, suggests that there is only minimal, if any, increase in delta-5-desaturase activity, and likely no increase at all in delta-4-desaturases in LE AT (for a review on poly-unsaturated fatty acids see [31]). With no other overt or generalized indications of increased poly-unsaturated lipogenesis in LE AT, indeed, no BH significant differences, these data might better interpreted to represent a situation of small-scale but localized inflammation i.e. involving the overproduction of C20:4 arachidonic acid. Thus, put simply, a localized and/or chronic increase in arachidonic acid, might lead to the compensatory biosynthetic increase and/or accumulation of anti-inflammatory omega-3-type lipids C20:5 and C22:6, the former appearing to being incorporated into TAG-type lipids, and both recently shown to be anti-inflammatory to human adipocytes [55,56]. Furthermore, we also found increased levels of ceramides in LE AT oil, especially Cer(d18:1/28:3) and HexCer(d18:1/23:0), which were increased up to 1.44-fold in LE. This might be particularly noteworthy, because these were by far the greatest magnitude increase in lipid-based molecules differentially present in this analysis. Thus, while we acknowledge that these are preliminary ceramide designations based solely on the mass/charge ratios (m/z values) these findings are biologically interesting for several reasons. For example, tissue ceramides are reported to be differentially affected by environmental oxygen levels—again supporting the idea of oxidative stress within LE AT, consistent with previous reports by other investigators [57]. Also, ceramides are down-stream metabolites of C20:4 arachidonic acid and other lipid-based secondary messengers that often associated with pro-inflammatory cytokines, such as tumor necrosis factor (TNF)[58]. These data might therefore reasonably reflect an inflammatory pathology in LE that involves TNF, i.e. such as through TNF-Receptor activated sphingomyelinases. (For review describing TNFR signaling pathways and inflammation see [59]). Moreover, this signature of inflammation is consistent with a recent transcriptomics analysis in human LE skin biopsy [60] and observations in the mouse tail model of experimentally-induced LE [61,62]. A thorough and cell-specific investigation of the cytokines produced by human AT leukocytes will be necessary to determine if and how TNF is integral to the production of C20:4 arachidonic acid, and ceramides, in human LE limb AT, and these studies are currently underway in our laboratory. Other pro-inflammatory cytokines such as IL-6 appear to also be important in both inflammation and adipose tissue pathobiology in LE [63]. Lastly, the increase in arachidonic acid and ceramides might additionally imply that an active process of cell death is occurring, e.g in inflammatory foci, in advanced LE AT. If true, then it is also immediately evident that nothing is known at present about the triggers of cell death in LE. While TNF could theoretically be involved, it is also possible that adipocyte death occurs independent of TNF. Indeed, auto-inflammatory pathways such as TLR- or inflammasome- triggered signaling events might be active in LE AT. In this regard are findings are consistent with recent studies which have shown that endogenous oils from human adipocytes are potent adjuvants, capable of promoting production of IL-1-alpha and inflammation [64], and with the observation that cholesterol can be a trigger for inflammasome and cell death, including the activation of macrophages forming crown-like structures, in other human lipid-rich tissue pathologies such as non-alcoholic steatohepatitis [65]. Further studies will be required to determine the exact mechanisms of inflammation in human advanced LE AT.

Also of interest, this lipidomic analysis verifies an earlier report of the presence of cyclopropane-containing fatty acids in human serum and AT [66]. While these types of fatty acid molecules have long been known to exist in plants, bacteria and protozoans, their origins in humans are much less clear and to our knowledge this is only the second report of branched-chain fatty acids in human AT [66]. It is possible that their low abundance in human serum and AT may simply reflect their original metabolism in plants i.e. after entering humans via dietary consumption—similar to many omega-3 fatty acids. Alternatively, their presence in human AT potentially reflects their synthesis within the human microbiome of the host, for example, such as from the individuals’ intestinal *E*.*coli*, *Lactobacillus spp*., and/or other microbial flora [67], consistent with emerging evidence that certain gut microbial metabolites may accumulate in host adipose tissue lipids [68]. It is also theoretically possible that cyclopropane-type fatty acids are endogenously produced within human adipocytes themselves, however, their endogenous production by human cells has not been demonstrated to date. Independent of their origin, the biological significance of their accumulation in human serum and AT is currently unknown.

Furthermore, with respect to the branched-chain fatty acids and cyclopropane-containing fatty acids, they are known to have significant impact when present within membranes. These types of lipids can have extraordinary effects on the rigidity and fluidity of plasma membranes [69]; their abundance correlates with biophysical properties of lipid membranes, including transition temperature, phase, and ultimately thereby to cell shape and cell plasticity, etc. The degree and position of branching within a lipid species also influences the volume needed for each lipid molecule within the membrane lipid array [70], and thus, also, to lipid packing and lipid bi-layer thickness [69]. So too, changes in the extent of fatty acid saturation can affect the functionality of a membrane. It is therefore tempting to speculate on the small but statistically significantly elevated levels of MHA observed in LE AT oil. This could be either biologically completely inconsequential (not above background variation and normal sample heterogenicity), or alternatively, it might indicate that there are small physiological tolerances for adipocyte membrane fluidity, plasticity, and/or phospholipid bilayer thickness, and hence that the small but statistically significantly elevated abundance of MHA presence could contribute to adipocyte pathology in LE. Indeed, that the MHA increase was evident in AT oil (but not intact AT fat) might reflect selective rupture of the MHA-high adipocyte membranes. In vitro experiments that introduce these types of lipids into experimental membranes may help to determine their biophysical effects on membrane fragility and function, and support (or disprove) these hypotheses.

One of the values of this type of clinical study is that it quickly draws attention back to the patients themselves, and in this case, the physical impact of living with LE. Hence we note again that it was clear that most of the changes were arguably rather unexpectedly subtle and this was despite the long-established (sometimes life-long) extreme limb enlargement that occurs in individuals living with primary or cancer related LE (see Fig 1). It is therefore important to carefully consider whether these extremely small but statistically different changes in specific lipids represents a genuine biochemical imbalance or pathology in AT, or more simply, a condition that reflects the effects of physiological limitations that occur in LE, i.e. impaired limb function due to swelling. In either case, the chronicity of the clinical condition means that differences, although individually small, may be especially biologically significant over time. For example, even small changes in membrane phospholipids, such as sphingomyelin chain length or cholesterol type and abundance, can influence membrane lipid-raft mobility and the functions of membrane embedded proteins—especially for glycosyl-phosphatidylinositol (GPI)-anchored membrane-associated [71,72] and ion channel proteins [73]. These changes, especially in the levels of MHA and highly saturated lipids, might therefore cumulatively alter adipocyte membrane function and/or fragility. If true, then together with the number of years of living with LE (showing a positive statistical association; see Table 2) and the physical effects of constant frequent lymphatic massage may explain the accumulation of AT oil in swollen LE tissue. Indeed, even if the changes documented here only affect a small number of adipocytes (or other AT cells), such as might occur in foci of inflammation, nevertheless, the chronicity of the clinical condition still likely exerts biological significance in the affected individual.

Finally, we documented that there was no evidence of hyperlipidemia in advanced LE, yet an individuals’ weight and body mass index (BMI) has is associated with LE occurrence [74,75]. This is true both for primary LE as well as severity of secondary or cancer-related LE [76,77]. However, whether “weight” is correlative or causal in human LE incidence, however, has been difficult or near impossible to confidently define. Interestingly, the muscle-related lymphatic contractile pump capacity is emerging as a potential factor in the etiology of LE [78]. Recent reports of *in vivo* mouse studies have shown that when wild type mice are fed a high-fat diet, inducing hypercholesterolemia, there is reduced collecting lymphatic vessel contractile capacity [79]. While these mice did not develop lymphedema per se, the increase in tissue adiposity impacted negatively on lymphatic vessel function [79]. Furthermore, in apoE-/- mice, which spontaneously develop hypercholesterolemia, there is evidence of decreased lymphatic drainage and spontaneous tissue swelling i.e. spontaneous murine LE [80]. Given our evidence of relatively normal lipid biochemistry in LE AT, coupled with the findings that adiposity effects on lymphatic vessel contractile capability, suggest that weight control and moderate exercise regimes remain important components to be considered for the successful long-term management of LE—even in the absence of hyperlipidemia in these patients. This study therefore provides important new information concerning LE AT which can be added to the current body of information on the pathobiology of human lymphatic disease [81].

In summary, this study provides the first comprehensive evidence showing that LE AT is essentially normal with respect to the tissue lipidome and that there appears to be a subtle inflammatory signature in LE AT, especially within the LE AT oil. This leads us to caution patients and LE clinicians to avoid tissue stresses that could further damage intact adipocytes thereby causing the liberation of more adipocyte oil. Indeed, the presence of the abundant free AT oil, with an increased pro-inflammatory signature comprising arachidonic acid and ceramides, appears to identify the existence of an underlying pathology in advanced LE AT–even though the AT lipidome itself is otherwise essentially normal. Hence, our data, in the context of other published studies, may help to explain why reducing LE AT burden (even though the LE AT is largely normal AT), for example, through liposuction surgery, appropriate exercise and a well-managed carefully chosen low-fat diet, is providing a relatively successful long-term management strategy for patients with primary and cancer-related LE [9–12]. Further research is underway in our laboratory to better characterize the LE associated AT inflammation.

## Supporting Information

S1 Data File Fatty Acids. Adipose tissue & serum fatty acid lipodemic data analysis.
(XLSX)

S2 Data File DAGs & TAGs. Adipose tissue diacylglycerides (DAGs) & triacylglycerides (TAGs) lipodemic data analysis.
(XLSX)

S3 Data File Phospholipids. Adipose tissue phospholipid lipodemic data analysis.
(XLSX)

S1 Fig Principal Component Analysis (PCA). Adipose tissue (A) Fatty acids, (B) DAGs & TAGs, and (C) Phospholipids.
(TIFF)

## Abbreviations

AT: adipose tissue
BMI: body mass index
Cer: ceramide
CE: cholesterol ester
DAG: diacylglyceride
GC: gas chromatography
HexCer: hexa-ceramide
HDL: high density lipoprotein
LE: lymphedema
LC: liquid chromatography
LDL: low density lipoprotein
LPC: lyso-phosphatidylcholine
LPE: lyso-phosphatidylethanolamines
PBQC: pooled biological quality control
PC: phosphatidylcholine
PCA: principle component analysis
PE: phosphatidylethanolamine
MS: mass spectrometry
SM: sphingomyelin
TAG: triacylglyceride
VLDL: very low density lipoproteins
