# Supplementary figures and images for: Lipidomic Profiling of Adipose Tissue Reveals an Inflammatory Signature in Cancer-Related and Primary Lymphedema
Source: PLoS One. 2016 May 16;11(5):e0154650. doi: 10.1371/journal.pone.0154650 (PMC4868287; doi:10.1371/journal.pone.0154650)

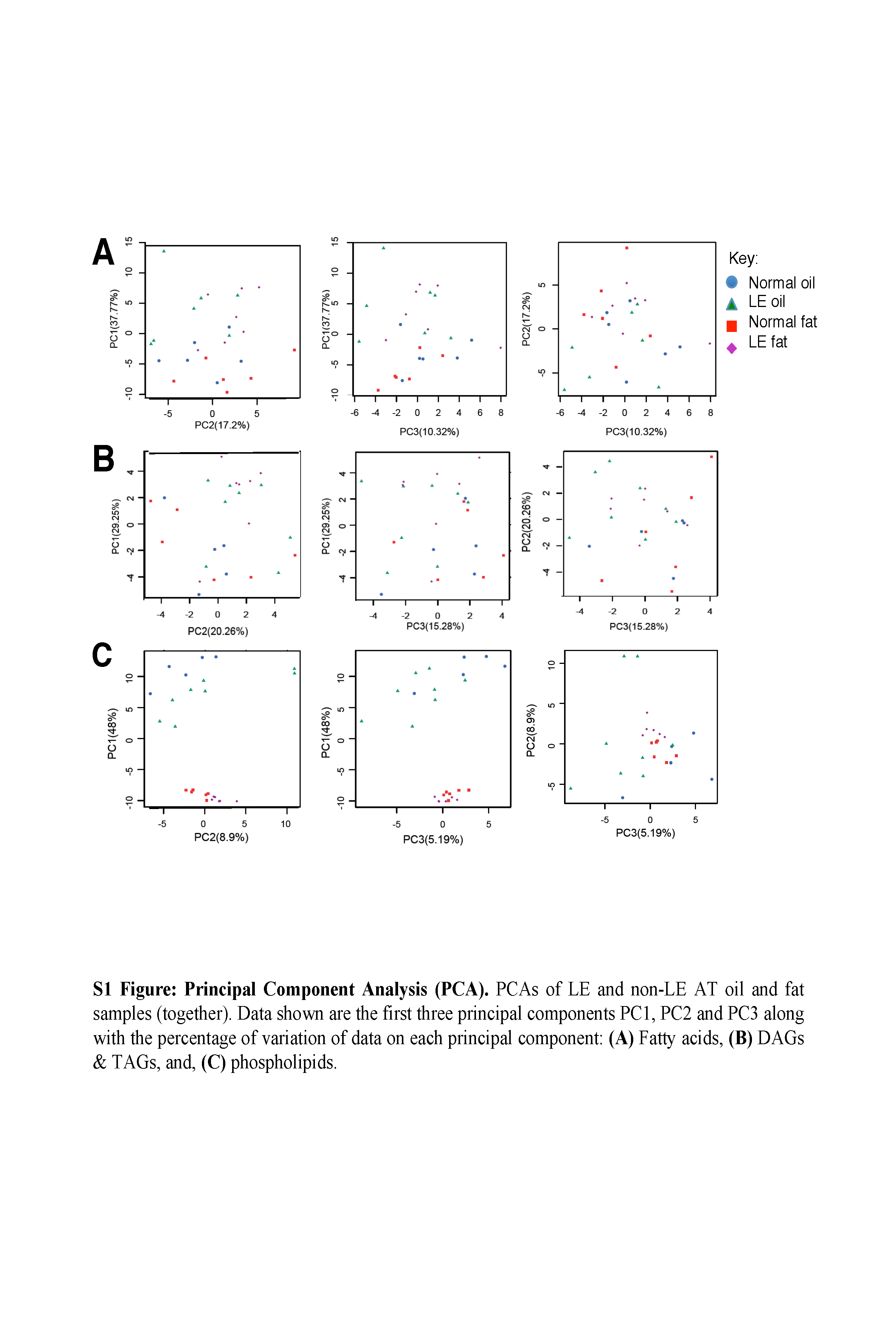

Supplement: S1 Fig — Adipose tissue (A) Fatty acids, (B) DAGs & TAGs, and (C) Phospholipids. (TIFF) [file pone.0154650.s004.tiff]
